# Diabetes Mellitus and Poorer Prognosis in Hepatocellular Carcinoma: A Systematic Review and Meta-Analysis

**DOI:** 10.1371/journal.pone.0095485

**Published:** 2014-05-15

**Authors:** Yan-Gang Wang, Peng Wang, Bin Wang, Zheng-Ju Fu, Wen-Juan Zhao, Sheng-Li Yan

**Affiliations:** Department of Endocrinology, the Affiliated Hospital of Medical College, Qingdao University, Qingdao, China; Nanjing Medical University, China

## Abstract

**Background:**

Previous studies suggested that diabetes mellitus was associated with cancer risk and prognosis, but studies investigating the relationship between diabetes mellitus and survival in patients with hepatocellular carcinoma (HCC) reported inconsistent findings. To derive a more precise estimate of the prognostic role of diabetes mellitus in HCC, we systematically reviewed published studies and carried out a meta-analysis.

**Methods:**

Eligible articles were identified in electronic databases from their inception through September 16, 2013. To evaluate the correlation between diabetes mellitus and prognosis in HCC, the pooled hazard ratios (HR) and their 95% confidence intervals (95% CI) for poorer overall and disease-free survivals were calculated by standard meta-analysis techniques with fixed-effects or random-effects models.

**Results:**

21 studies with a total of 9,767 HCC patients stratifying overall survival and/or disease-free survival in HCC patients by diabetes mellitus status were eligible for meta-analysis. 20 studies with a total of 9,727 HCC cases investigated the overall survival, and 10 studies with a total of 2,412 HCC patients investigated the disease-free survival. The pooled HRs for overall survival and disease-free survival were 1.46 (95% CI, 1.29 to 1.66; P<0.001) and 1.57 (95% CI, 1.21 to 2.05; P = 0.001), respectively. The adjusted HRs for overall survival and disease-free survival were 1.55 (95% CI, 1.27 to 1.91; P<0.001) and 2.15 (95% CI, 1.75 to 2.63; P<0.001), respectively. In addition, for patients receiving hepatic resection, diabetes mellitus was associated with both poorer overall survival and poorer disease-free survival, and for patients receiving non-surgical treatment or patients receiving radiofrequency ablation, diabetes mellitus was associated with poorer overall survival. There was no evidence for publication bias.

**Conclusion:**

Diabetes mellitus is independently associated with both poorer overall survival and poorer disease-free survival in HCC patients.

## Introduction

Hepatocellular carcinoma (HCC) is one of the most common malignancies and a major cause of death among both sexes, and despite diagnostic and therapeutic improvements, its incidence and mortality rates have obviously increased in recent years, especially in Asian countries [1], [2]. Although the survival of HCC patients has been improved by advances in surgical techniques and perioperative management, such as radiofrequency ablation (RFA) and transcatheter arterial chemoembolization (TACE), long-term survival remains unsatisfactory owing to the high rate of recurrence and metastasis [3], [4]. To guide decision-making for therapeutic strategies for HCC patients and improve their prognosis, a better understanding of the relevant factors affecting HCC prognosis is urgently needed. Some independent prognostic factors for survival, such as age and liver function, have already been identified and are useful when choosing the best treatment on an individual status [5], [6]. Diabetes mellitus is a common disease that has a tremendous impact on human health worldwide, and epidemiologic evidence suggests that people with diabetes are at significantly elevated risk of many kinds of cancer, such as pancreatic, lung, colorectal and gastric cancers [7]–[10]. There is also some epidemiologic evidence suggesting that diabetes mellitus is associated with poorer prognosis in cancer patients, but previous studies investigating the relationship between diabetes mellitus and survival in HCC patients have reported inconsistent findings [11]–[14]. To derive a more precise estimate of the prognostic significance of diabetes mellitus in HCC patients, we systematically review published studies and carried out a meta-analysis by using standard meta-analysis techniques (Checklist S1). We followed the Meta-analysis of Observational Studies in Epidemiology (MOOSE) consensus in this systematic review and meta-analysis [15].

## Materials and Methods

### Search Strategy

We conducted a comprehensive literature search in Pubmed, Embase, Web of Science, Ovid, Google Scholar, and Chinese Biomedical Database (CBM) databases from their inception through September 16, 2013. We combined search terms for diabetes mellitus and HCC: (“liver cancer” or “hepatocellular carcinoma” or “hepatic cancer”) and (“diabetes mellitus” or “diabetes” or “glucose intolerance” or “hyperglycemia”). There was no language limitation. All references cited in those included studies were also reviewed to identify additional published articles not indexed in the common databases.

### Study Eligibility

We included studies that evaluated the association of diabetes mellitus with overall survival (OS; date of surgery to date of death as a result of any cause) and disease-free survival (DFS; date of surgery to date of first recurrence or death), and the diabetes mellitus diagnosis were based on the definitions described by the World Health Organization or the American Diabetes Association. Only full papers and published studies in the medical literature were included. Data from abstracts, review articles, editorials, case reports, and letters were not included. We excluded studies for which no hazard ratio (HR) with its 95% confidence interval (95% CI) could be calculated for any of the outcomes. Discrepancies were resolved by a consensus in regular meetings attended by at least three-quarters of the investigators. In case of multiple publications from the same institution with identical or overlapping patient cohorts, only the most informative publication was included.

### Data Extraction

Quality assessment for cohort studies in this meta-analysis was assessed using the Newcastle Ottawa scale (NOS) as recommended by the Cochrane Non-Randomized Studies Methods Working Group [16]. This instrument was developed to assess the quality of nonrandomized studies, specifically cohort and case-control studies. This scale awards a maximum of nine stars to each study: four stars for the adequate selection of cohort participants, two stars for comparability of cohort participants on the basis of the design and analysis, and three stars for the adequate ascertainment of outcomes [16]. Given the variability in quality of observational studies found on our initial literature search, we considered studies that met 5 or more of the NOS criteria as high quality.

### Statistical Analysis

We calculated the pooled HR with its corresponding 95% CI to assess the associations of diabetes mellitus with OS and DFS, and an HR greater than 1 indicated a worse prognosis in patients with diabetes mellitus. The significance of the pooled HR was determined by the Z test and a P value of less than 0.05 was considered significant. In our study, two models of meta-analysis for dichotomous outcomes were conducted: the random-effects model and the fixed-effects model [17], [18]. The random-effects model was conducted using the DerSimonian and Laird’s method, which assumed that studies were taken from populations with varying effect sizes and calculated the study weights both from in-study and between-study variances [18]. The fixed-effects model was conducted using the Mantel-Haenszel’s method, which assumed that studies were sampled from populations with the same effect size and made an adjustment to the study weights according to the in-study variance [17]. To assess the between-study heterogeneity, the I^2^ statistic to quantify the proportion of the total variation due to heterogeneity was calculated [19]. The I^2^ index expressing the percentage of the total variation across studies due to heterogeneity was calculated to assess the between-study heterogeneity, and I^2^ values of >50% suggested high heterogeneity [19]. If high heterogeneity existed, the random-effects model was used to pool the results; otherwise, the fixed-effects model was used to pool the results when I^2^ value was less than 50%. For additional analyses, meta-analyses were subgrouped on the basis of their analysis styles (multivariate analyses or univariate analyses) and treatment methods (hepatic resection, non-surgical treatment, or RFA). Because characteristics of participants were not consistent between studies, we further conducted meta-regression analysis to explore possible explanations for heterogeneity if high heterogeneity existed [20]. To validate the credibility of outcomes in this meta-analysis, sensitivity analysis was performed by sequential omission of individual studies or by omitting studies without high quality [21]. Potential publication bias was assessed by visual inspection of the funnel plots, in which the standard error of logor of each study was plotted against its logor, and an asymmetric plot suggested possible publication bias. In addition, we also performed Egger linear regression test at the P<0.05 level of significance to assess the funnel-plot’s asymmetry [22]. All analyses were performed using STATA version 12.0 (Stata Corp, College Station, TX, USA). A P value<0.05 was considered statistically significant, except where otherwise specified.

## Results

### Study Characteristics

**Figure 1:**
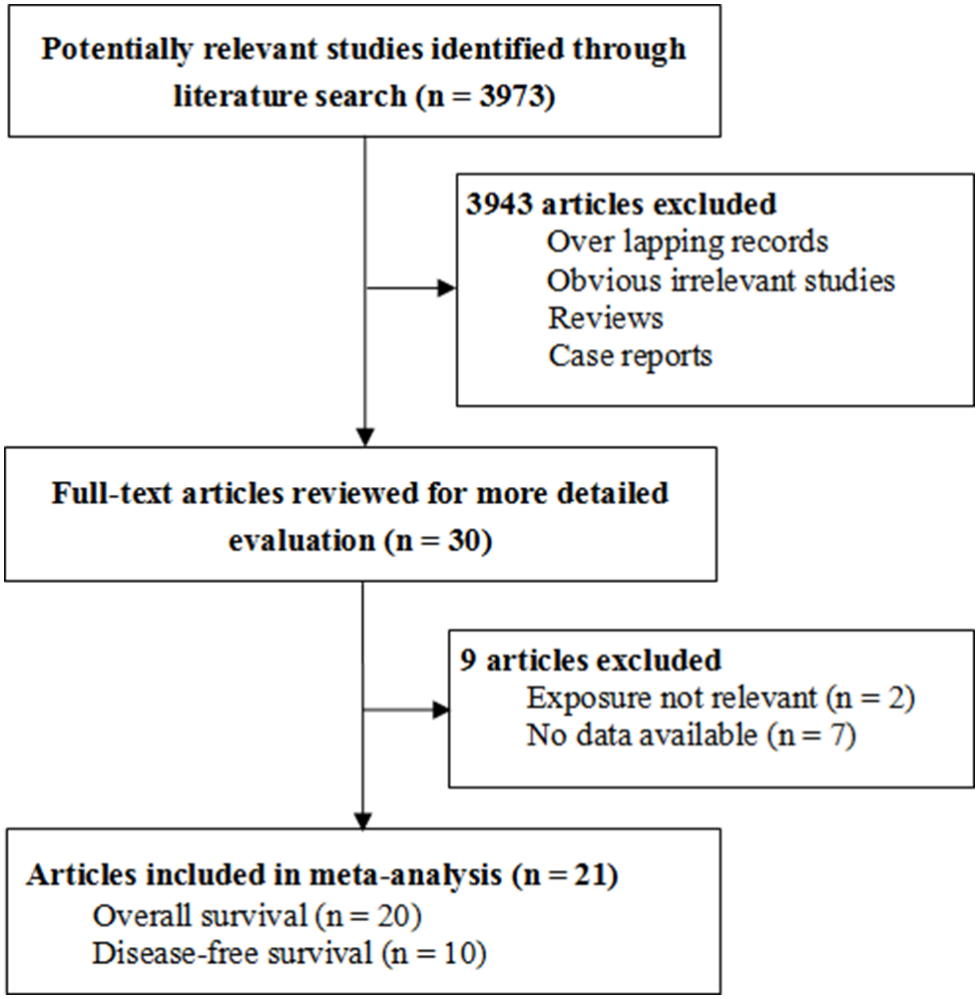
Flow chart of study selection in this systematic review.

Figure 1 illustrated the process of evaluating articles for inclusion in the review and meta-analysis. Of the 3,973 abstracts identified, we excluded 3,943 abstracts and further reviewed 30 full-text articles to determine whether they met our inclusion and exclusion criteria [23]–[52]. 8 studies were excluded for no data available [43], [45]–[48], [50]–[52], and 2 studies were excluded for irrelevant studies [44], [49]. One article included two different patient cohorts, and was extracted as two separate studies [42]. Thus, 21 studies from 20 articles with a total of 9,767 HCC patients stratifying OS and/or DFS in HCC patients by diabetes mellitus status were included in the meta-analysis [23]–[42]. The main characteristics of the 21 eligible studies are shown in Table 1. The total number of included patients was 9,767, ranging from 40 to 2815 patients per study (median: 465). 20 studies with a total of 9727 HCC cases investigated the OS, and 10 studies with a total of 2412 HCC patients investigated the DFS (Table 1). According to the quality criteria, there were 18 studies with high quality, and 3 studies with low quality (Table 1).

**Table 1:** Main characteristic of 21 eligible studies in this meta-analysis.

Study authors	Recruitment time	Patients (Diabetes mellitus percent)	Follow up (median time)	Outcomes^§^	Quality scores
Yanaga K 2003 [41]	Between April 1985 and July 1990	209 HCC patients treatedwith hepatic resection (23.4%)	5.5 years	OS	5
Ikeda Y 1998 [29]	Between April 1985 and March 1995	342 HCC patients treatedwith hepatic resection (25.4%)	1,278 days	OS^†^; DFS^†^	8
Toyoda H 2001 [40]	Between 1990 and 1999	581 patients with HCC treatedwith various methods (15.8%)	32 months	OS; DFS	6
Poon RT 2002 [36]	Between 1989 and 2000	525 HCC patients treatedwith hepatic resection (11.8%)	54 months	OS; DFS	7
Li XP 2003 [32]	From January 1998 to December 2001	225 patients withunresectable HCC (12.4%)	3 years	OS	4
Huo TI 2003 [28]	Between 1996 and 1999	239 HCC patients treatedwith hepatic resection (16.3%)	32 months	OS	5
Huo TI 2004 [42]	From April 1996 to March 2001	255 HCC patients whounderwent surgical resection(16.1%)	33 months	OS^†^	7
Huo TI 2004 [42]	From April 1996 to March 2001	312 patients withunresectable HCC (25.3%)	33 months	OS^†^	7
Park SM 2006 [35]	From 1996 to 2002	2815 patients with HCCtreated with various methods (10.5%)	3.03 years	OS^†^	7
Komura T 2007 [31]	Between June 1987 and May 2004	90 HCC patients treated withhepatic resection (33.3%)	5 years	OS; DFS^†^	7
Sumie S 2007 [38]	Between January 1994 and December 2000	120 patients with HCC treatedwith various methods (33.1%)	57 months	OS; DFS	5
Kawamura Y 2008 [30]	From 1980 to December 2006	40 HCC patients treated withhepatic resection (45.0%)	5.7 years	DFS^†^	4
Huo TI 2010 [27]	Prospectively evaluated starting from 2002	1713 patients with HCCtreated with various methods(22.9%)	18 months	OS^†^	7
Chen WT 2011 [24]	From 2004 to 2007	161 patients with HCC treatedwith RFA (32.9%)	3 years	OS; DFS	5
Feng YH 2011 [25]	From August 2007 to June 2008	52 patients with HCC treatedwith TACE (26.9%)	18 months	OS; DFS^†^	5
Chen TM 2011 [23]	Between July 2003 and June 2009	114 patients with HCC treatedwith RFA (28.1%)	3 years	OS^†^; DFS^†^	5
Howell J 2011 [26]	Between January 2000 and August 2007	135 patients with HCC treatedwith various methods (43.0%)	5 years	OS^†^	5
Shau WY 2012 [37]	Between 2003 and 2004	931 patients with HCC treatedwith various methods (19.9%)	62.8 months	OS^†^	7
Ting CT 2012 [39]	Between January 2000 and December 2008	389 HCC patients treated with hepatic resection (30.1%)	5 years	OS^†^; DFS†	6
Ou DP 2007 [34]	From 1992 to 2005	446 HCC patients treated withhepatic resection (8.1%)	58 months	OS	5
Liu XY 2010 [33]	From 2002 to 2008	75 patients with HCC treatedwith various methods (33.1%)	3 years	OS	3

(^†^data from multivariate analysis; ^§^OS was for overall survival, while DFS was for disease-free survival; HCC, hepatocellular carcinoma; RFA, radiofrequency ablation; TACE, transcatheter arterial chemoembolization).

### Meta-analysis

**Figure 2:**
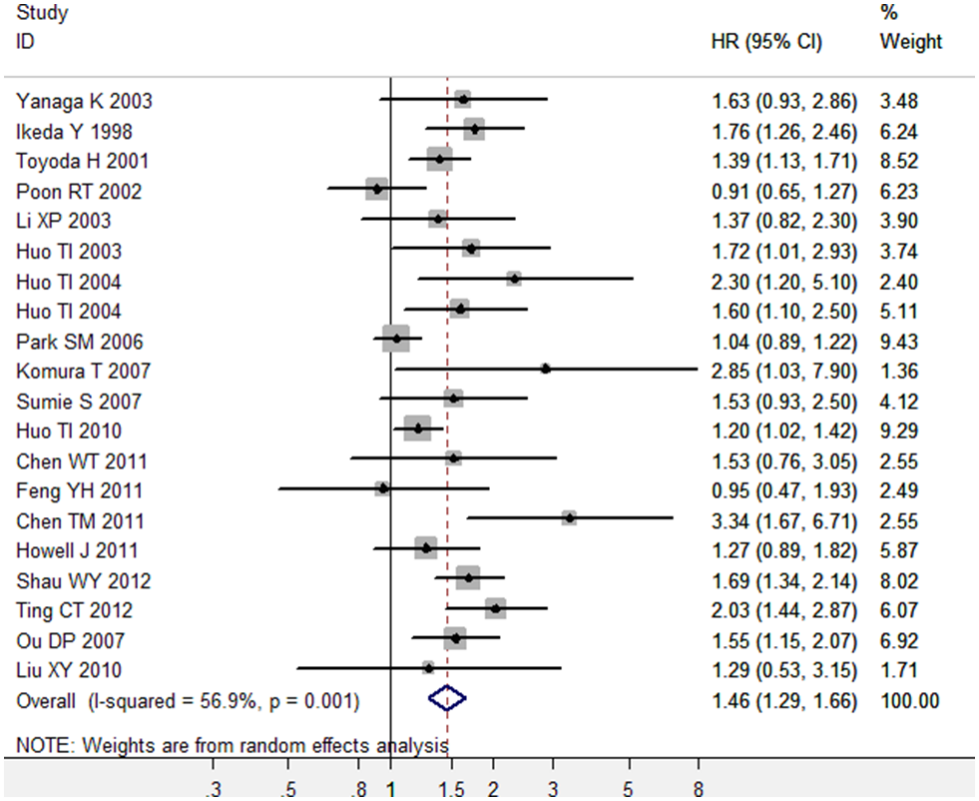
Meta-analysis of the association between diabetes mellitus and overall survival in HCC.

Of the 20 studies about OS, there was obvious between-study heterogeneity (I^2^ = 56.9%), thus the random-effects model was used to pool the results. The pooled HR for OS was 1. 46 (95% CI, 1.29 to 1.66; P<0.001) (Figure 2, Table 2). Sensitivity analysis by sequential omission of individual studies or by omitting studies without high quality didn’t alter the significance of combined HR estimate, which validated the credibility of outcomes. Meta-regression analysis showed that treatment method was the possible explanation for heterogeneity (P<0.05). Subgroup analyses by multivariate analyses or univariate analyses showed the combined HR estimate for OS under multivariate analyses was 1.55 (95% CI, 1.27 to 1.91; P<0.001), while the combined HR estimate for OS under univariate analyses was 1.37 (95% CI, 1.21 to 1.55; P<0.001) (Table 2). Subgroup analyses by treatment methods suggested diabetes mellitus is associated with poorer overall survival in HCC patients received hepatic resection (P<0.001), non-surgical treatment (P<0.001) and RFA (P<0.001) (Table 2).

**Table 2:** Results of meta-analysis of the association between diabetes mellitus and prognosis in HCC.

Endpoint analyzed	Studies(Patients)	HR (95% CI)^†^	P value	Heterogeneity^*^
**Overall survival**				
Total studies	20(9,727)	1.46(1.29–1.66)	<0.001	56.9%
Subgroup-multivariate analyses	9(7,006)	1.55(1.27–1.91)	<0.001	75.8%
Subgroup-univariate analyses	11(2,721)	1.37(1.21–1.55)	<0.001	7.6%
Subgroup-Hepatic resection	9(3,426)	1.64(1.35–2.00)	<0.001	49.7%
Subgroup-Nonsurgical treatment	6(1,795)	1.65(1.31–2.08)	<0.001	33.7%
Subgroup-RFA	3(1,206)	2.19(1.51–3.18)	<0.001	18.7%
**Disease-free survival**				
Total studies	10(2,412)	1.57(1.21–2.05)	0.001	78.1%
Subgroup-multivariate analyses	6(1,027)	2.15(1.75–2.63)	<0.001	23.8%
Subgroup-univariate analyses	4(1,385)	1.06(0.94–1.20)	0.346	0.0%
Subgroup-Hepatic resection	6(1,027)	1.91(1.21–3.00)	0.005	84.0%
Subgroup-Nonsurgical treatment	3(327)	2.30(0.75–7.00)	0.143	78.6%
Subgroup-RFA	2(275)	1.70(0.50–5.75)	0.393	78.6%

(^†^HR (95% CI), hazard ratio with its 95% confidence interval; *The value of I^2^ for Heterogeneity).

**Figure 3:**
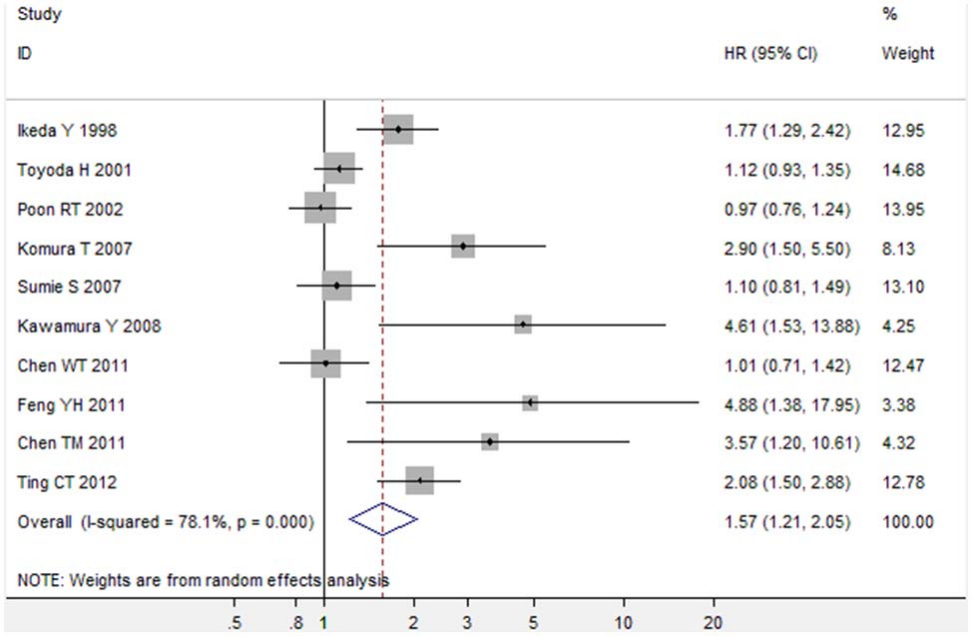
Meta-analysis of the association between diabetes mellitus and disease-free survival in HCC.

Of the 10 studies about DFS, there was also obvious between-study heterogeneity (I^2^ = 78.1%), thus the random-effects model was used to pool the results. The pooled HR for DFS was 1.57 (95% CI, 1.21 to 2.05; P = 0.001) (Figure 3, Table 2). Sensitivity analysis by sequential omission of individual studies or by omitting studies without high quality didn’t alter the significance of combined HR estimate, which validated the credibility of outcomes. Meta-regression analysis further showed that analysis style (multivariate analyses or univariate analyses) was the possible explanation for heterogeneity (P<0.01). Subgroup analyses by multivariate analyses or univariate analyses showed the combined HR estimate for DFS under multivariate analyses was 2.15 (95% CI, 1.75 to 2.63; P<0.001) (Table 2). Subgroup analyses by treatment methods suggested diabetes mellitus is associated with poorer DFS in HCC patients received hepatic resection (P = 0.005).

### Publication Bias

Funnel plot and Egger’s test were performed to assess the publication bias in the meta-analysis. Funnel plots’ shape of all contrasts did not reveal obvious evidence of asymmetry, and all the P values of Egger’s test were more than 0.05, providing statistical evidence of funnel plots’ symmetry. Thus, the results above suggested that publication bias was not evident in this meta-analysis.

## Discussion

Both diabetes mellitus and liver cancer are global problems with devastating human, social and economic impact, and growing evidence shows that there is an obvious relationship between diabetes mellitus and increased risk of HCC [4], [9], [53]. Though previous meta-analysis by Wang C et al. provides strong evidence for the association between diabetes mellitu and risk of HCC incidence, there is no direct evidence for the association between diabetes mellitu and survival in HCC patients [53]. It’s no doubt that identifying the prognostic markers of HCC can guide clinical decision-making in the treatment of HCC, and improve the patients’ prognosis. Previous studies prove that diabetes mellitus is an independent prognostic factor for several common human malignancies, such as breast cancer, colorectal cancer, and prostate cancer [10]–[13]. Diabetes mellitus may be a promising prognostic marker to predict poorer survival in HCC, and there are also many studies investigating the prognostic value of diabetes mellitus in patients with HCC, but it remains uncertain because of the inconsistent findings from available publications [23]–[42].

In current meta-analysis, studies reporting HRs of cumulative survival rates were summarized qualitatively by using standard meta-analysis techniques. 21 studies with a total of 9,767 HCC patients stratifying OS and/or DFS by diabetes mellitus status were eligible for meta-analysis. The pooled HRs for OS and DFS were 1.46 (95% CI, 1.29 to 1.66; P<0.001) and 1.57 (95% CI, 1.21 to 2.05; P = 0.001), respectively. When the analysis was restricted to multivariate analyses, we also observed a statistically significant detrimental effect of diabetes mellitus on the survival of HCC patients, suggesting diabetes mellitus was an independent prognostic factor for HCC. In addition, for patients receiving hepatic resection, diabetes mellitus was associated with both poorer OS and poorer DFS, and for patients receiving non-surgical treatment and patients receiving RFA, diabetes mellitus was associated with poorer OS. Thus, diabetes mellitus is independently associated with both poorer OS and poorer DFS in patients with HCC.

The role of diabetes mellitus in hepatocarcinogenesis has been widely studied, and there are several biologic mechanisms standing for the prognostic role of diabetes mellitus in HCC [54]–[57]. Diabetes may influence the recurrence of HCC by hyperinsulinemia, hyperglycemia, or chronic inflammation [54]–[57]. Insulin may work directly on epithelial cells or indirectly by activating insulin-like growth factor pathways or altering endogenous sex hormones, and insulin resistance appears to play a key role in HCC recurrence [54]–[57]. Hyperinsulinemia related to underlying insulin resistance is associated with an increasing growth rate of cancer cells which may play important roles in the progression of HCC. In addition, free radicals caused by oxidative stress and chronic inflammation in diabetes patients may also be able to promote the progression and metastasis of HCC. Thus, there is some biologic plausibility for the prognostic role of diabetes mellitus in HCC patients.

The meta-analysis suggests diabetes mellitus is independently associated with both poorer OS and poorer DFS in HCC patients. To improve the prognosis of HCC patients with diabetes, it is time for integrated thinking and action to erode the large overlapping burden between these two diseases. Though improved glucose control remains one of the central goals of effective diabetes management, several factors should be considered by clinicians and HCC patients when selecting pharmacologic diabetes therapies [58], [59]. Current studies suggest there are different effects on risk of cancer among different pharmacologic diabetes therapies [58]–[62]. Insulin use is more strongly associated with increased risk of overall, pancreatic, and colorectal cancer, while metformin and thiazolidinediones areassociated with a lower risk of overall, liver, and colorectal cancer [23], [25], [58]–[62]. For patients with both HCC and diabetes mellitus, metformin and thiazolidinediones may be better choices than insulin use [60]–[62]. However, there are also lots of other antidiabetes drugs, and it’s still unclear which the best choice is for patients with both HCC and diabetes mellitus. In the future, more well-designed randomized controlled trials or prospective cohort studies are urgently needed to capture a better knowledge on the choice of antidiabetes drugs for HCC patients.

Compared with previous studies, our meta-analysis has several strengths. Firstly, previous studies didn’t include all eligible studies (one was only 8 studies, and the other was 10 studies) and could inevitably increase the risk of bias [63], [64]. Our meta-analysis includes 21 eligible studies with a total of 9,767 HCC patients stratifying OS and/or DFS in HCC patients by diabetes mellitus status, which provide a stronger statistical power and a more precise estimation. In addition, pervious studies didn’t perform subgroup analysis by the adjusted a status of HRs, but our meta-analysis provided the pooled HRs of adjusting for other potential confounders, and concluded that diabetes mellitus was an independent prognostic factor for HCC. Finally, subgroup analyses by the treatment methods were also preformed in our meta-analysis, which was not discussed in previous studies. The finding from the subgroup analyses further identified the prognostic role of diabetes mellitus in HCC patients receiving different treatments. These strengths above all provide a stronger evidence for the prognostic role of diabetes mellitus in HCC patients.

There were also several limitations to be considered when interpreting the findings in our meta-analysis. Firstly, the HRs calculated in our meta-analysis could be overestimated as a result of reporting biases because many studies were retrospective cohort studies. Thus, adequately designed prospective studies with an appropriate multivariate analysis taking into account the classical well-defined prognostic factors for HCC are needed to get a more precise estimate on the prognostic role of diabetes mellitus in HCC. Secondly, there were only three studies on HCC patients receiving RFA treatment, and the limited studies could inevitably increase risk of random error. Thus, more studies with large sample sizes are needed to further identified the prognostic role of diabetes mellitus in HCC patients receiving RFA treatment. Finally, the included studies did not report the types of diabetic therapy used or their impact on outcomes. This is important because previous studies have shown that some therapies may have a negative impact on cancer outcomes, whereas others may be beneficial [23], [25], [58]–[62]. Additional well-conducted and appropriately designed prospective observational studies are needed to explore how specific diabetic therapies influence HCC prognosis.

In conclusion, diabetes mellitus is independently associated with both poorer overall survival and poorer disease-free survival in HCC patients. More well-designed randomized controlled trials or prospective cohort studies are urgently needed to explore how specific antidiabetes drugs influence the prognosis of HCC patients.

## Supporting Information

Checklist S1 **PRISMA Checklist.**
(DOC)
